# Characterization and antagonistic potentials of selected rhizosphere *Trichoderma* species against some *Fusarium* species

**DOI:** 10.3389/fmicb.2022.985874

**Published:** 2022-10-03

**Authors:** Olumayowa Mary Olowe, Lidia Nicola, Micheal Dare Asemoloye, Akinlolu Olalekan Akanmu, Ayodele Adegboyega Sobowale, Olubukola Oluranti Babalola

**Affiliations:** ^1^Food Security and Safety Focus Area, Faculty of Natural and Agricultural Sciences, North-West University, Private Mail Bag, Mmabatho, South Africa; ^2^Department of Earth and Environmental Sciences, Laboratory of Mycology, University of Pavia, Pavia, Italy; ^3^School of Pharmaceutical Science and Technology, Tianjin University, Tianjin, China; ^4^Department of Botany, Faculty of Science, University of Ibadan, Ibadan, Oyo State, Nigeria

**Keywords:** bioagents, pathogen, mycoparasitism, rhizosphere, culture filtrates, antagonism

## Abstract

*Trichoderma* fungi have been proved as efficient bioagents with great antifungal properties while many species in the plant’s rhizospheres have been characterized as plant growth-promoting agents. However, many rhizosphere *Trichoderma* are yet to be fully explored for plant disease management. In this study, *Trichoderma* species were isolated from the rhizosphere of maize, banana, and cassava, and their biocontrol potentials were screened against some *Fusarium* species from oak leaves (F2B and F3) and laboratory cultures (Fus 296 and Fus 294). The isolated rhizosphere *Trichoderma* were identified as *Trichoderma virens* 1 (TCIV), *T. virens* 2 (TCVII), *T. virens* 3 (TMSI), *T. hazianum* strain 1 (TCVI), *T. harzianum* strain 2 (TCVIII), *T. erinaceum* (TMZI), and *T. koningiopsis* (TMZII). The dual culture experiment recorded the highest percentage inhibition in TMZII against OakF2B (31.17%), TCVIII against Fus 294 (45.18%), TMZI against Fus 296 (47.37%), while TCIV was most effective against Oak F3 (44.15%). Among the *Trichoderma* culture filtrates evaluated, TCIV showed the highest percentage inhibition against Oak F3 (52.39%), Oak F2B (48.54%), Fus 294 (46.65%), and Fus 296 (44.48%). All the *Trichoderma* isolates demonstrated expressed varying levels of antagonism against the *Fusarium* pathogens *in vitro.*

## Introduction

Plant diseases are associated with diverse economic losses in various agricultural products and they presently constitute one of the most severe threats to food security and sustainability (Viana et al., 2022). The yield losses caused by pests and disease on food crops are enormous, resulting in 13–22% annual yield loss in staple crops such as wheat, rice, potato, and maize (Adeniji and Babalola, 2018). These are mostly caused by fungi and often result in poor quality and low yields (Nguyen et al., 2017; Olowe et al., 2020). *Fusarium* species are one of the causal agents of diseases in some crops of agricultural importance, such as rice and oak which are of major concern in Italy. *Fusarium*s species are known for their global distribution and economic significance as producers of toxins such as fumonisin and other compounds causing contamination in plants. They are soil-borne pathogens that cause severe damping-off, vascular wilts, and rots in crops of economic importance (Bodah, 2017). Also, stunted seedlings, necrosis, defoliation, and browning of xylem vessels are associated with *Fusarium* infections in plants which may ultimately lead to death. Compared to other soil-borne pathogens, *Fusarium* species infect a wide range of host plants on fruits, roots, leaves, and stems. *Fusarium fujikuroi*, for example, has been found to cause stem and head rots and head blight in crops such as maize, rye, barley, wheat, and millet. Another is the instance of *Fusarium tricinctum*, which causes head blight in various cereal crops and leads to the accumulation of mycotoxin in the grains. Chemical fungicides are used to manage *Fusarium* diseases, although, the efficacy attained with these treatments is frequently less than predicted, and there is a need to restrict their use due to adverse environmental impacts (Shah et al., 2018). Also, excessive chemical use has been found responsible for the emergence of resistant races of *Fusarium* pathogens (Gupta et al., 2020). However, the biological control strategy is a viable alternative for fungal diseases. Moreover, it poses minimal or no negative effects on humans or the environment (Marutescu et al., 2017; Gupta et al., 2020).

The biocontrol method involves using bioagents, which can be achieved by introducing a prospective microbe or employing native microorganisms with antifungicidal potentials to curtail the growth and establishment of pathogens (Ajilogba et al., 2013; Köhl et al., 2019). Fungi as biocontrol agents are beneficial microbes that reduce the negative impacts of plant pathogens while enhancing plant growth. Aside from their biocontrol potentials, they also exhibit other benefits such as lowering the effects of abiotic and innate physiological stressors in plants and seeds, respectively. Furthermore, plants’ nitrogen utilization and photosynthetic efficiency are also improved. Therefore, the use of fungal biocontrol agents is an essential, economically sustainable, eco-friendly, and long-term strategy for plant disease (Ajilogba et al., 2013; Olowe et al., 2020; Rojas et al., 2020).

*Trichoderma* species are excellent biocontrol agents because of their diverse modes of action against plant diseases, including nutrient competition, mycoparasitism, and hydrolytic enzyme antibiosis (Filizola et al., 2019). The biocontrol potential of *Trichoderma* against *Fusarium* pathogens and other plant fungal pathogens has been widely researched. However, the use of *Trichoderma* in the sustainable management of diseases affecting crops has not gained wide recognition because the species used have not been characterized in many instances, and misidentification of the organisms often discourages its acceptability and its further deployment to farmers. In addition, there is limited information on *Trichoderma* efficacy against crop diseases isolated from various temperate zones. It is important to comprehend its effectiveness under various environmental conditions. The experiment aimed to examine the efficacy of certain *Trichoderma* strains in inhibiting the growth of some *Fusarium* species of oak and rice and to characterize them using molecular tools.

## Materials and methods

### Source of rhizosphere *Trichoderma* species and *Fusarium* species

A total of seven *Trichoderma* isolates, namely: TMS1, TMZ1. TMZ11, TCV1, TCV11, TCV111, and TCIV were obtained from soils collected from the rhizosphere of maize, banana, and cassava at the Students’ Farm of the University of Ibadan, Nigeria at Latitude 7.4433°N, and Longitude 3.9003°E. *Fusarium* isolates, *Fus* 296, *Fus* 294, were obtained from the culture collection of the Mycology Laboratory, Earth and Environmental Sciences, University of Pavia, Italy (45°11′06.5″N and 9°09′49.3″E), originally isolated from diseased rice plants, while F2B and F3 were isolated from lesions on oak leaves which were collected from Turbigo (Province Milano), Italy (Latitude: 45.5308°N Longitude: 8.7367°E). The diseased oak leaves were, collected between April and May, 2021, and brought to the laboratory for further analysis. The leaves were hand-picked from the tree showing symptoms of oak decline. *Trichoderma* isolates and *Fusarium* pathogens were cultured on Potato Dextrose Agar (PDA) and incubated at 28 ± 2°C for 7 days before bioassay.

### Morpho-dimensional and molecular identification

The fungi were cultured on Potato Dextrose Agar (PDA) Petri dishes and incubated at 25°C for 7 days. The growing fungi were then examined morpho-dimensionally under a light microscope, observing the mycelium and the different reproductive structures. Molecular characterization was then performed to confirm the morphological identification. The isolates were cultured in Potato Dextrose Broth (PDB) and placed in a rotary shaker at 180 rpm for 1 week at 28 ± 2°C until the mycelia were well developed. The supernatant was removed by vacuum filtration, the mycelia were collected. The fungal genomic DNA was extracted using the NucleoSpin Plant II by Macherey-Nagel (Bethlehem, PA, United States), and subjected to PCR amplification of the internal transcribed spacer (ITS) region of the ITS1-5.8S-ITS2 rDNA gene, as described by Daccò et al. (2020). The PCR reaction was performed on a Thermocycler Bio-Rad T100 in a 25 μl reaction mixture containing 1× DREAM Taq Green PCR MasterMix reaction buffer (Thermo Scientific, Pittsburg, PA, United States), 2.5 μl (10 μM) of each primer, 3 μl of DNA sample, and 4.5 μl of Nuclease Free water. The PCR program was as follows: denaturation by heating for 5 min at 95°C, then 35 cycles of 30 s at 95°C, 45 s at 50°C, and 1 min at 72°C, and a final elongation step for 10 min at 72°C. The primers used were ITS1 (5′-TCCGTAGG TGAACCTGCGG-3′) and ITS4 (5′ TCCTCCGCTTATTGATATGC-3′; Daccò et al. 2020). PCR products were purified with ExoSAP-IT (Applied Biosystems, Foster City, CA, United States) according to the manufacturer’s protocol. The amplified and purified DNA was sent to BMR Genomics (Padova, Italy), and the sequences were compared with target sequences using BLAST online^1^ and MEGA X 10.1.7.

### Phylogenetic analysis

The phylogenetic analysis was performed using the generated ITS rRNA gene sequences from all the fungal strains used in this work. The sequences of the isolated pathogenic strains were aligned with other sequences from *Fusarium* species previously documented on the NCBI using Cluster X 2.1 (Thomson et al., 1997). The phylogenetic relationship between these selected fungi with our strains was determined using the Molecular Evolutionary Genetics Analysis (MEGA 7.0) software (Kumar et al., 2016). The neighbor-joining tree was performed using the Kimura 2-parameter distances with bootstrapping of 1,000 replicates, while *Puccinia oxalidis* OK093305 isolated from the leaf of *Oxalis debilis* in India was used as an outgroup to root the tree. Similarly, the sequences of the isolated fungal bioagents were aligned with other sequences from *Trichoderma* species previously documented on the NCBI.

### Dual culture assay

**Figure 1:**
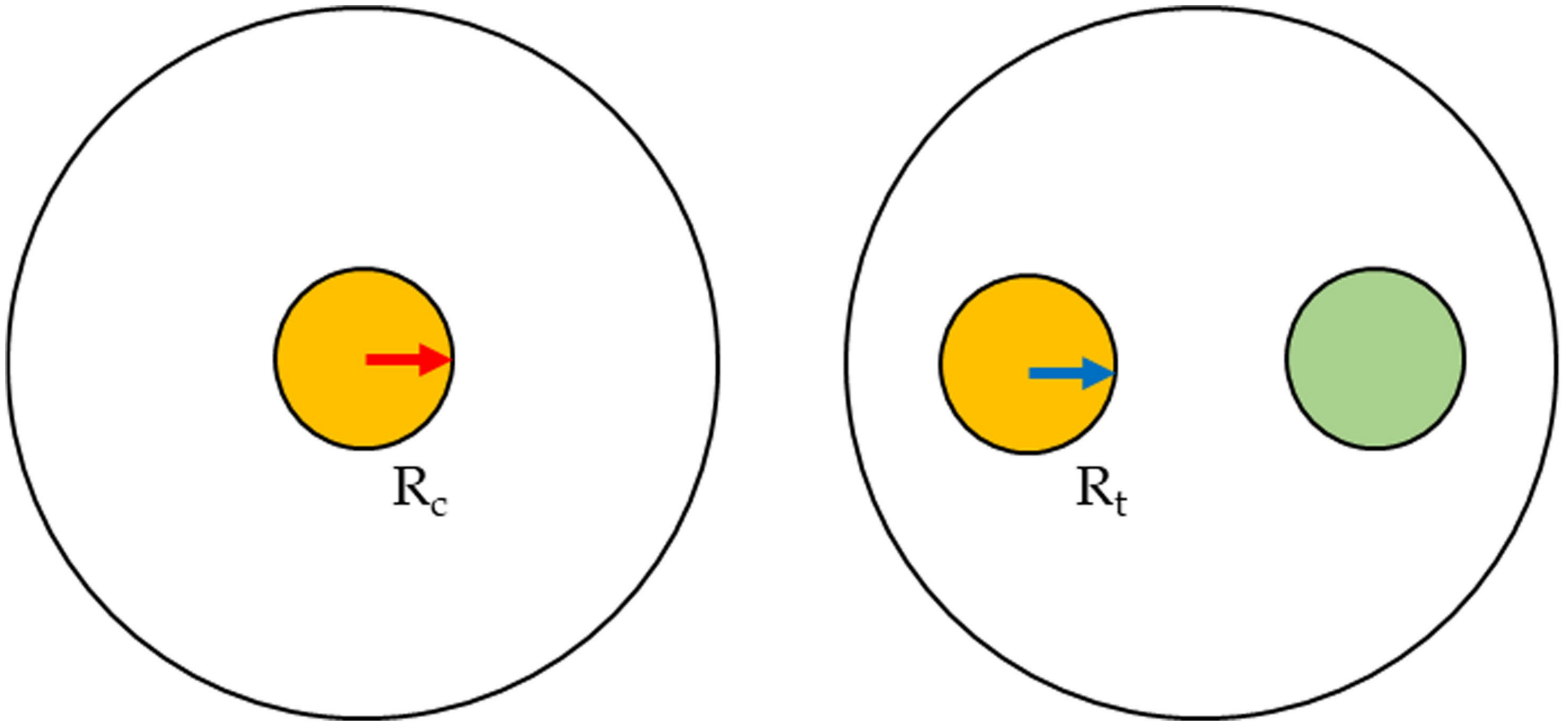
Inoculum scheme for the dual cultures.

The anti-fungi activity of seven *Trichoderma* strains was screened against the *Fusarium* spp. through dual culture techniques. PDA plates were each inoculated with mycelium plugs (5 mm diameter) from separate cultures of *Trichoderma* and *Fusarium*. The mycelium plugs were removed from the growing margins of the 1-week culture of the fungi and placed on the opposite sides of the 90 mm diameter of the Petri-dishes, at a distance of 4 cm (Figure 1). Each *Fusarium–Trichoderma* combination has three replicates. PDA plates were also inoculated only with the *Fusarium* species only to serve as the control. The plates were incubated at 25 ± 2°C for 7 days. The radius of the mycelial growth was observed and measured every 24 h until the pathogen had grown to cover the plate’s surface. The percentage inhibition rate was calculated as:


$$\%\mathrm{growth}=\frac{R_{c}-R_{t}}{R_{c}}*100$$

where:

*R_t_* = radius of the *Fusarium* when inoculated in a plate with *Trichoderma.*

*R_c_* = radius of the *Fusarium* strain when *inoculated* alone (control).

### Antifungal potency of culture filtrates of *Trichoderma* isolates

Five millimeters (5 mm diameter) mycelial plugs of the *Trichoderma* isolates from 3 to 5 days old culture were inoculated into 500 ml conical flasks, each containing 250 ml of sterilized PDB medium. The flasks were placed on the rotary shaker and incubated at room temperature with an agitation of 108 rpm for 1 week. After 1 week of incubation, the culture filtrates were collected into falcon tubes using a vacuum pump with sterilized paper filters. The culture filtrates were re-filtered twice using a sterile syringe filter of 0.2 μm. Five hundred microliter (500 μl) of the final fungal suspension were distributed on PDA plates using an L-shaped wand and were left to dry under the laminar flow hood. Each *Fusarium* species was inoculated on the plates containing the culture filtrate and without the culture filtrate (control). Three replicates were observed for each combination of *Fusarium*–*Trichoderma* culture filtrate.

### Statistical analysis

Data obtained were subjected to analyses of variance (ANOVA) using Statistical Analysis Software (SAS 2003), while means were separated using Duncan’s Multiple Range Test at *p* ≤ 0.05.

## Results

In this study, three fungi and seven bioagents were isolated, and identified using PCR-ITS amplification of the *rRNA* gene. Based on BLAST Search on the NCBI, the pathogens were identified as *Fusarium proliferatum* Strain 294, *Fusarium proliferatum* Strain 296, and *Fusarium tricinctum* Strain F2B. The bioagents were identified as *Trichoderma virens* Strain TCIV, *Trichoderma harzianum* Strain TCVI, *Trichoderma virens* Strain TCVII, *Trichoderma harzianum* Strain, *Trichoderma virens* Strain TMS1, *Trichoderma erinaceum* Strain TMZ1, and *Trichoderma koningiopsis* Strain TMZII (Table 1). The *Trichoderma* species were then documented on the NCBI under the accession numbers ON149743–ON149749.

**Table 1:** Identities of the *Fusarium* and the isolated *Trichoderma* species.

Strain code	Best BLAST match(es)	Accession code	Overlap length	Match (%)	Maximum score (%)
294	*Fusarium proliferatum*	MH857322.1	974	99.28	99.00
	*Fusarium fujikuroi*	KX772393.1	972	99.04	98.09
296	*Fusarium proliferatum*	MW425873.1	969	100.00	99.78
	*Fusarium fujikuroi*	MT229300.1	967	98.55	99.70
F2B	*Fusarium tricinctum*	MK102656.1	963	100	100.00
	*Fusarium babinda*	NR_159861.1	935	97.46	100.00
TCIV	*Trichoderma virens*	MT530036.1	998	99.00	99.40
	*Trichoderma virens*	KU666466.1	998	98.8	99.00
TCVI	*Trichoderma harzianum*	KF144639.1	959	100.00	100.00
	*Trichoderma harzianum*	KM100819.1	813	98.99	100.00
TCVII	*Trichoderma virens*	MT530036.1	1,021	99.67	100.00
	*Trichoderma virens*	MT529862.1	994	100.00	100.00
TCVIII	*Trichoderma harzianum*	MK738149.1	1,002	99.34	99.45
	*Trichoderma harzianum*	OL440920.1	973	99.12	99.00
TMS1	*Trichoderma virens*	MK517548.1	999	100.00	100.00
	*Trichoderma virens*	MT530036.1	995	100.00	100.00
TMZ1	*Trichoderma erinaceum*	MT186195.1	1,077	100.00	99.88
	*Trichoderma erinaceum*	MT186195.1	1,012	99.45	99.78
TMZII	*Trichoderma kkoningiopsis*	LC661385.1	897	99.34	99.98
	*Trichoderma koningiopsis*	LC661385.1	889	98.99	99.45

**Figure 2:**
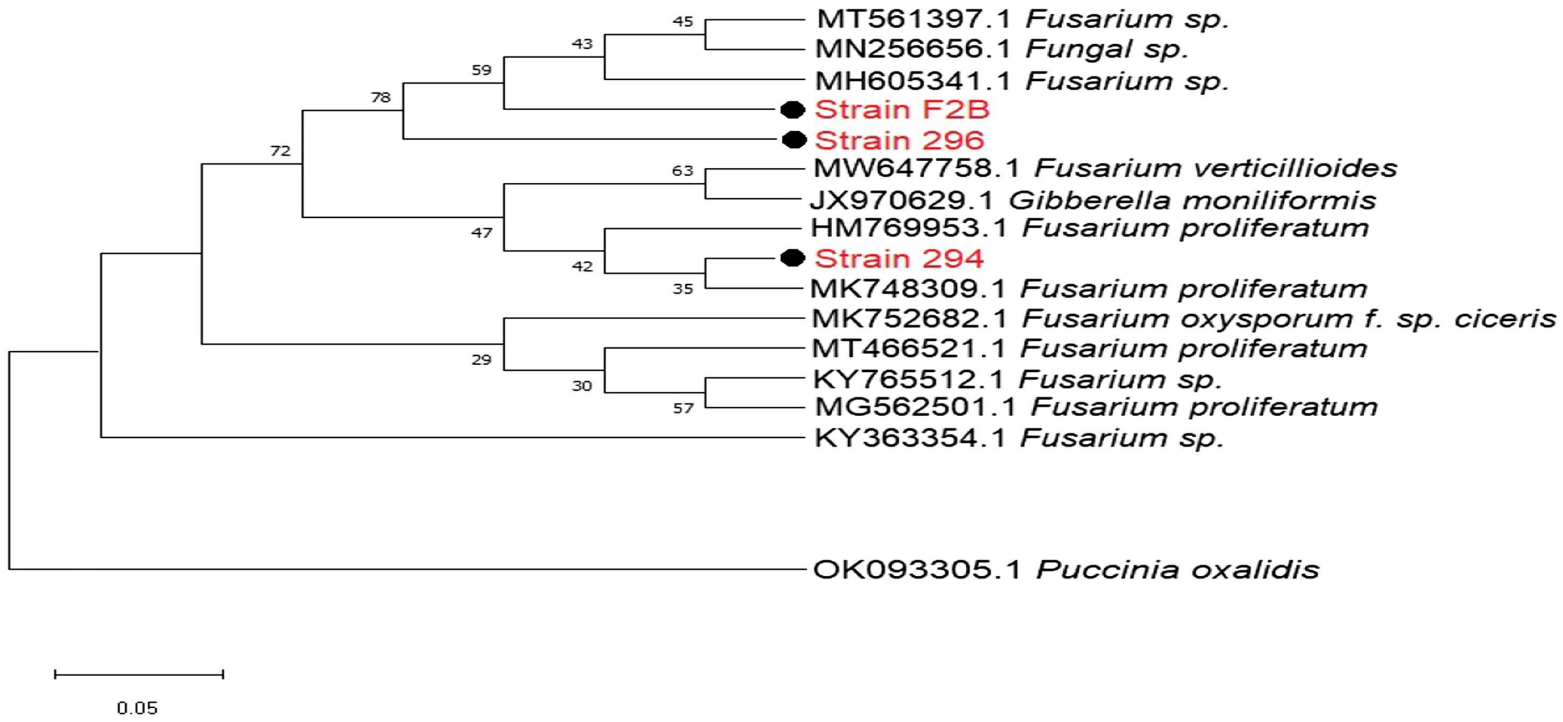
Phylogenetic relationships between the isolated *Fusarium*pathogenic strains and selected database relatives on the NCBI generated through internal transcribed spacer (ITS) rRNA gene analysed *via* Kimura’s two parameter models. Bootstrap support values higher than 50% from 1,000 replicates are shown at the nodes. *P. oxalidis* (OK093303) was used as the out-group.

The DNA sequences from isolated *Fusarium* strains were successfully aligned with other sequences of strains that were previously documented on the NCBI. The neighbor-joining tree revealed the phylogenetic relationships between the selected *Fusarium* pathogens (Figure 2). However, our isolated strain 294, clustered into a distinct well-supported clade with *F. proliferatum* HM769953 and three other pathogenic strains HM769953, JX970629, and MW647758 isolated from India (Figure 2).

**Figure 3:**
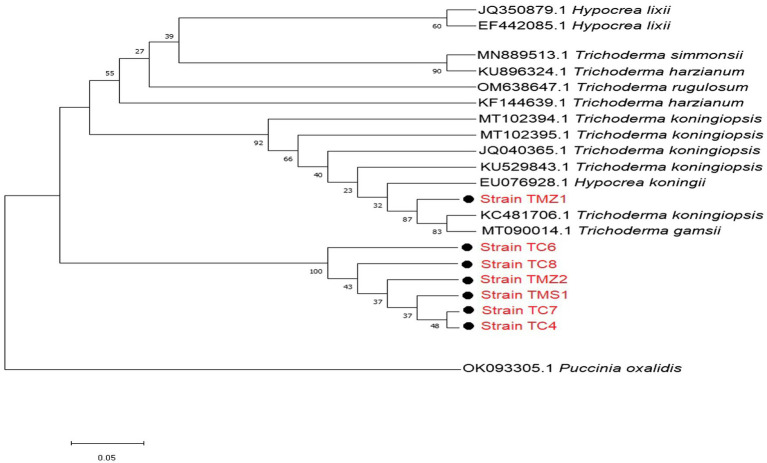
Phylogenetic relationships between the isolated bioagent strains and selected database of *Trichoderma* relatives from the NCBI analyzed *via* Kimura’s two-parameter models. Bootstrap support values higher than 50% from 1,000 replicates are shown at the nodes. *P. oxalidis* (OK093303) was used as the out-group.

For the *Trichoderma* strains, most of our strains except TMZ1 clustered into a distinct well-supported clade with each other. They all showed more similar phylogenetic relationship with each other than those that were previously documented on the NCBI (Figure 3). Our two strains; *T. virens* TCIV and TCVII showed the most similar phylogenetic relationship while both clustered into another related clade with TMS1, TMZII, TCVIII, and TCVI (Figure 3).

**Figure 4:**
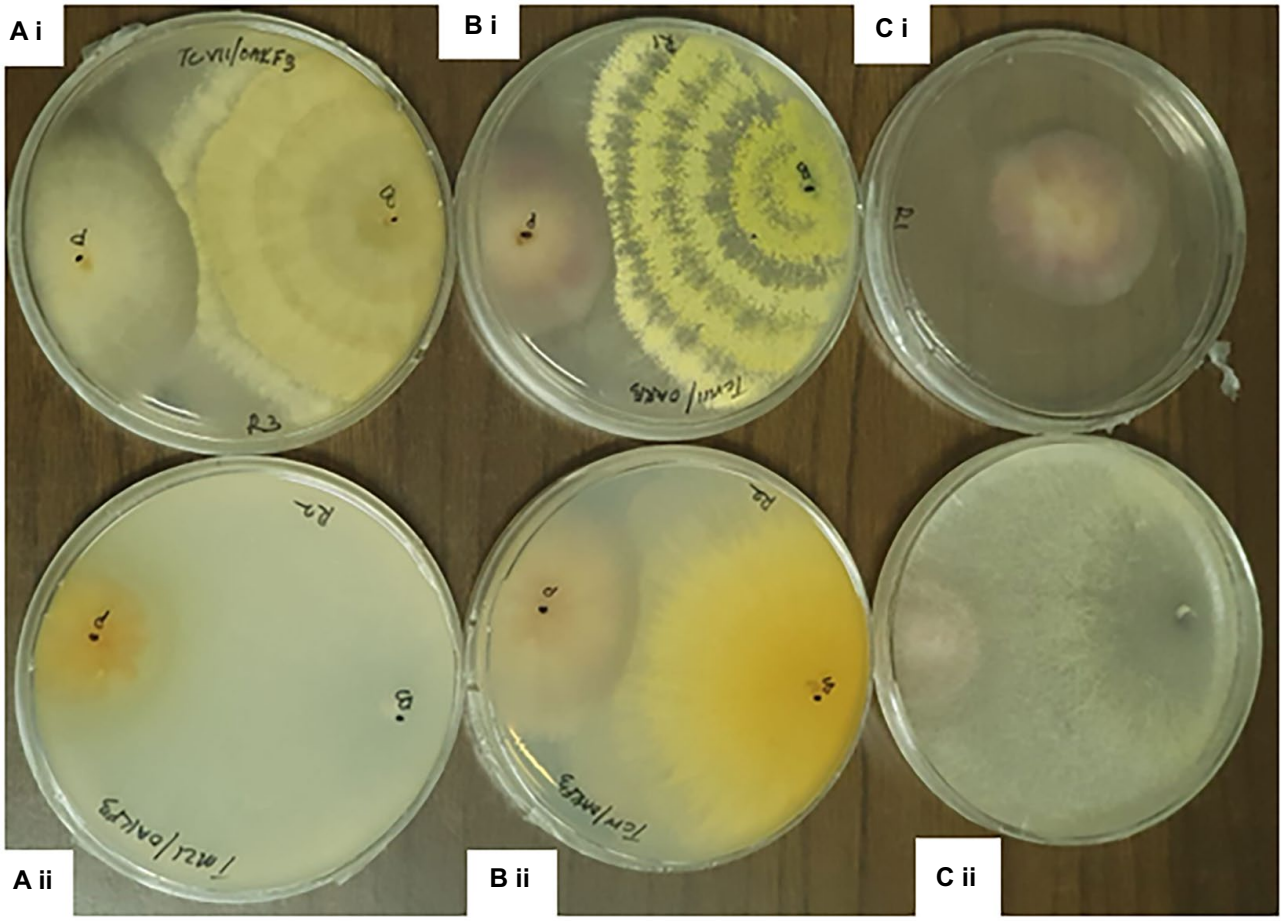
Dual culture assay of *Trichoderma* species against *Fusarium* pathogens at 7th day of Incubation. **ai**= TCVII/ OakF3, **bi**= TCVIII/OakF3, **ci**= Control **aii**= TMZI/ Oak F3, **bii**= TCIV/OakF3, **cii**= TMZII/OakF3.

**Figure 5:**
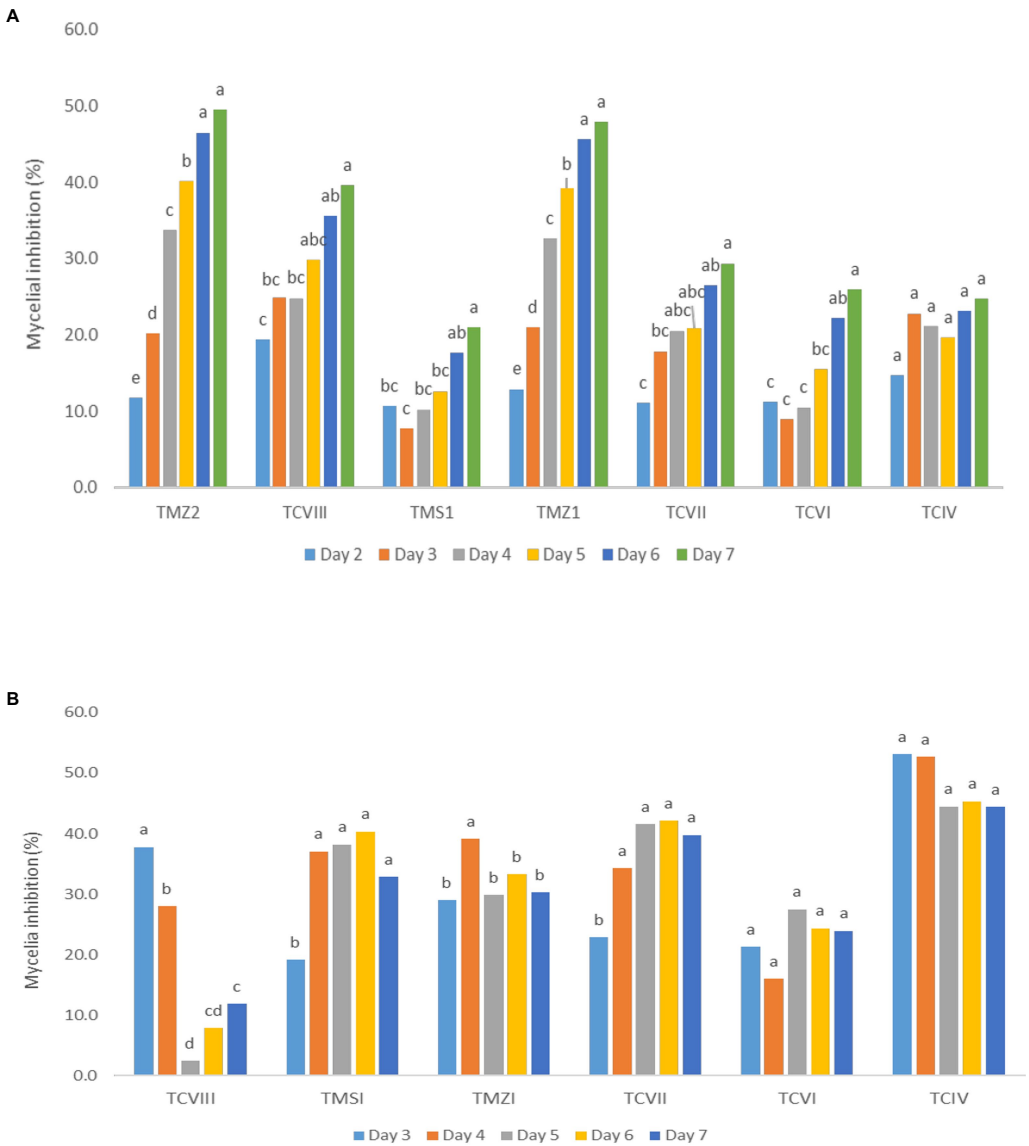
Inhibitory potentials of *Trichoderma* species through **(A)** the dual culture technique, and **(B)** culture filtrates, against *Fusarium* species with respect to days of observation. Means with different letters are significantly (*p* < 0.05) different across each *Trichoderma* treatment.

**Figure 6:**
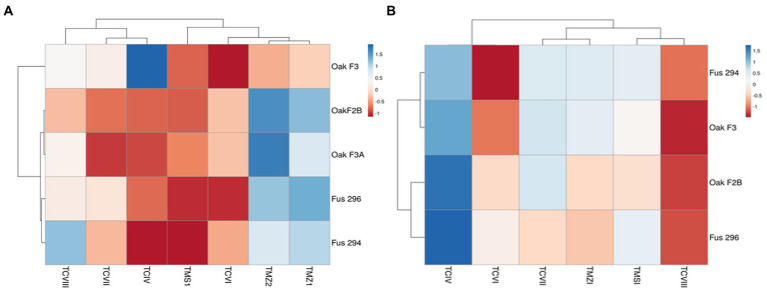
Pooled antagonistic effect of *Trichoderma* species against *Fusarium* pathogens with **(A)** dual culture, and **(B)** culture filtrates.

The percentage inhibition of *Fusarium* pathogens (OakF2B, Fus 296, Fus 294, Oak F3, Oak 3FA) by the antagonistic *Trichoderma* species (TMZ1, TMZII, TMS1, TCIV, TCVI, TCVII, and TCV8) in the dual culture experiment showed significant (*p* < 0.05) inhibition as examined after 7^th^ day of observation in the dual culture (Figure 4). All the antagonistic *Trichoderma* species increased percentage inhibition with increasing days of evaluation on all the *Fusarium* species tested, except in TCIV against Oak F3, which was most inhibited at day 4 (71.26%; Table 2; Figure 5A). The highest percentage inhibition was recorded in TMZII against OakF2B (31.17%), TCVIII against Fus 294 (45.18%), TMZI against Fus 296 (47.37%), while TCIV was most effective against Oak F3 (44.15%). Furthermore, the pooled effect of *Trichoderma* species in this study showed TMZII > TMZI > TCVIII > TCVII > TCVI > TCIV > TMS1 as the order of antagonistic efficacy against the *Fusarium* species, while Fus 294 > Fus 296 > OakF3A > OakF2B > Oak F3 (Figure 6A).

**Table 2:** Percentage inhibition (%) of the *Fusarium* species by mycelia of the *Trichoderma* isolates in dual culture

Organisms	Days	TMZII	TCVIII	TMS1	TMZ1	TCVII	TCVI	TCIV
OakF2B	2	6.25c	8.61c	0.00d	10.69d	4.17a	2.08e	4.31b
	3	14.47c	10.37c	7.40c	13.35d	10.10a	10.30de	5.75b
	4	28.48b	12.07c	9.31c	22.75c	12.93a	15.03 cd	8.03b
	5	40.52a	21.84b	20.83b	36.60b	22.79a	23.65bc	24.75a
	6	47.27a	30.76a	28.92a	43.83ab	26.51a	32.29ab	27.21a
	7	50.00a	34.12a	32.50a	46.67a	25.83a	35.83a	30.00a
	LSD	10.79	6.56	6.25	9.31	21.13	10.35	7.12
Fus 296	2	15.39d	33.33a	10.26ab	20.51e	12.82c	20.51a	20.51ab
	3	29.17c	12.87b	0.36b	32.19d	27.42bc	9.24a	11.32ab
	4	47.92b	24.47ab	12.15ab	50.09c	21.92c	12.36a	17.29ab
	5	50.46b	25.31ab	6.64b	52.93bc	27.62abc	5.40a	6.33b
	6	58.99a	38.25a	17.29ab	61.17ab	32.15ab	10.17a	17.03ab
	7	65.38a	48.75a	28.06a	67.34a	45.79a	16.99a	28.01a
	LSD	7.94	22.48	19.57	10.13	17.99	18.3	18.24
Fus 294	2	16.05c	21.69c	15.93a	18.02e	7.84b	18.02 cd	12.01ab
	3	28.29b	47.98ab	4.29bc	29.65d	24.12ab	11.49de	8.74b
	4	35.79b	37.94b	1.24c	40.87c	29.41ab	8.74e	2.57b
	5	47.07a	50.02ab	12.62ab	51.41b	19.68ab	25.07bc	11.27ab
	6	52.84a	55.75a	17.41a	56.74ab	27.12ab	32.30ab	18.99a
	7	55.69a	57.73a	21.22a	59.27a	31.79a	36.25a	18.49a
	LSD	8.27	12.8	8.57	7.48	20.64	8.08	8.87
Oak F3	2	0.00d	0.00a	0.00b	0.00b	0.00b	0.00b	0.00c
	3	7.15c	25.21a	8.18ab	17.81a	17.80a	0.00b	69.70a
	4	13.61b	27.59a	11.26a	18.05a	27.13a	1.15b	71.26a
	5	13.59b	31.29a	10.59a	15.43a	26.05a	0.00b	51.26ab
	6	20.57a	26.72a	10.11a	22.10a	28.99a	6.89a	40.98ab
	7	21.84a	27.86a	5.04ab	20.09a	26.05a	8.38a	31.69bc
	LSD	5.63	32.38	8.25	12.69	13.17	4.87	35.16

Means with different letters are significantly (*p* < 0.05) different across the column in each section. LSD: least significant difference.

**Figure 7:**
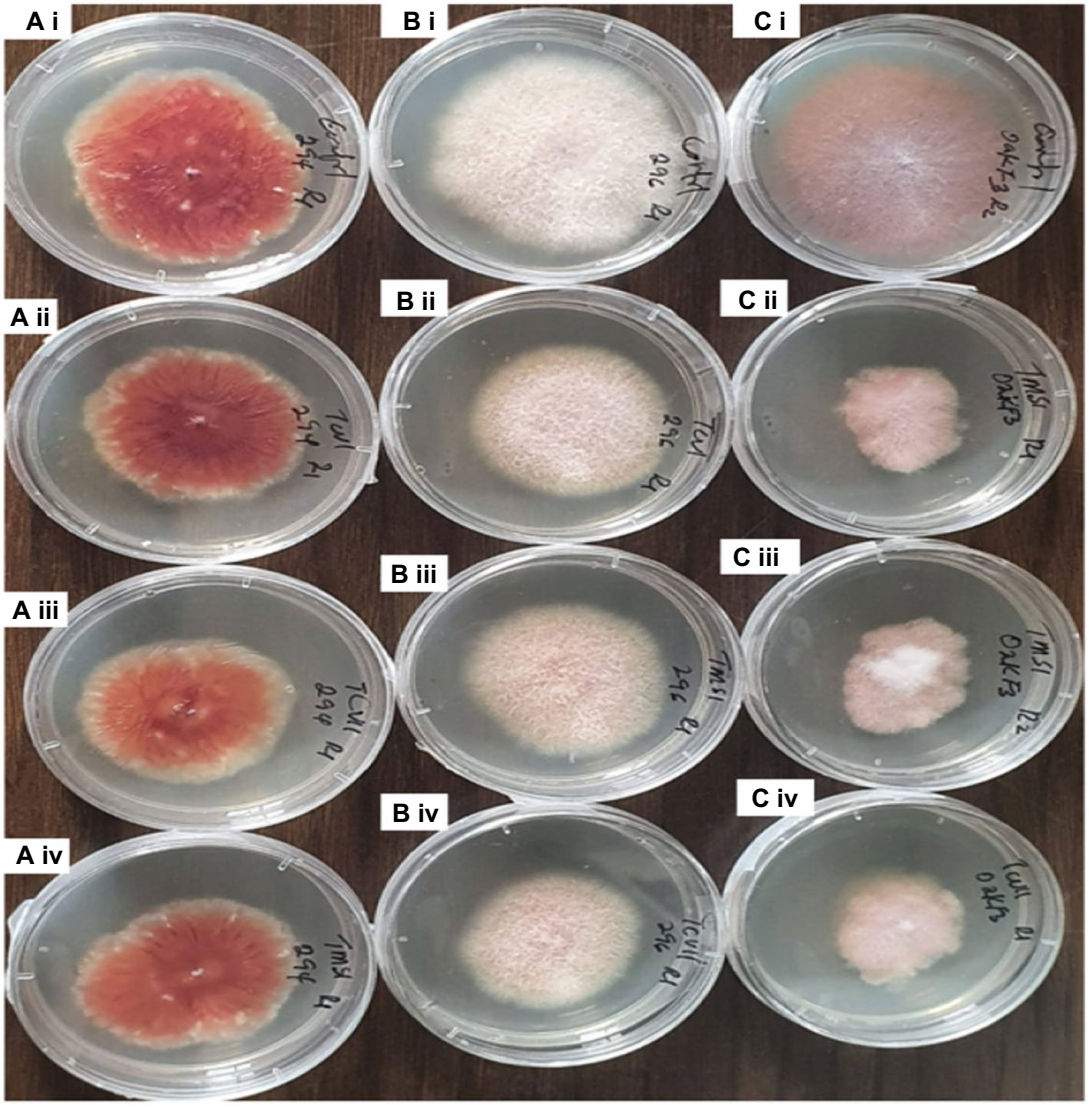
Inhibitory effect of the culture filtrates of antagonistic isolates of *Trichoderma* against plant pathogens *Fusarium* on 7th day of observation. **Ai**= Fus 294 (control), **Bi**= Fus 296 (control), **Ci**= OakF3, **Aii**= TCVI/Fus294, **Bii**= TCVI/Fus 296, **Cii**= TMZI/ OakF3 (RI), **Aiii**= TVCII/Fus294, **Biii**= TMSI/Fus296, **Ciii**= TMZI/ OakF3 (R2), **Aiv**= TMSI/Fus294, **Biv**= TCVII/Fus296, **Civ**= TCVII/OakF3.

The results obtained from the *Trichoderma* culture filtrate showed increasing percentage inhibition of TCIV, TMSI, and TCVII across the *Fusarium* species while others showed effectiveness against selected pathogens at varying days (Table 3; Figures 5B, 7). Generally, TCIV showed the highest percentage inhibition against Oak F3 (52.39%), Oak F2B (48.54%), Fus 294 (46.65%) and Fus 296 (44.48%). The pooled result showed TCIV > TCVII > TMSI > TMZI > TCVI > TCVIII as the order of inhibition by the culture filtrates. Also, this study showed OakF3 > OakF2B > Fus 294 and Fus 296 as the inhibited pathogens (Figure 6B).

**Table 3:** Percentage inhibition (%) of the *Fusarium* species by culture filtrates of the *Trichoderma* isolates.

Organism	Days	TCVIII	TMS1	TMZ1	TCVII	TCVI	TCIV
OakF2B	3	38.76a	12.80b	42.47a	20.67b	6.06b	52.23a
	4	39.15a	48.38a	42.05a	49.06a	6.67b	53.85a
	5	3.922b	43.04a	34.21a	40.77a	14.93ab	44.85a
	6	5.36b	42.48a	38.14a	46.08a	20.00a	44.34a
	7	4.35b	44.89a	38.14a	41.77a	13.78ab	37.96a
	LSD	6.42	12.24	11.56	11.66	9.49	17.49
Fus 296	3	30.00a	13.33b	26.67b	36.67a	13.33c	43.33a
	4	20.87ab	25.75ab	33.87a	37.13a	18.11bc	54.75a
	5	2.08c	29.58a	23.33b	40.28a	40.56a	44.58a
	6	8.18bc	36.03a	24.60b	34.44a	27.94abc	47.46a
	7	23.00ab	31.99a	22.95b	37.07a	33.27ab	52.57a
	LSD	15.28	15.03	7.16	24.85	18.09	17.2
Fus 294	3	32.22a	28.89a	6.11c	2.78c	26.11ab	52.22a
	4	21.67b	32.36a	39.03a	0.00c	34.86a	49.72a
	5	3.81c	32.03a	22.77b	41.51a	26.25ab	43.36a
	6	7.94c	31.75a	22.22b	36.51a	23.81ab	39.68a
	7	6.78c	24.97a	16.57c	31.81b	20.63b	37.43a
	LSD	4.36	15.44	11.15	6.43	11.83	16.32
Oak F3	3	50.00a	21.52c	40.91a	31.21b	39.39a	64.85a
	4	30.26b	41.71ab	41.71a	50.94a	4.44a	52.48a
	5	0.00d	48.06a	39.17a	43.47a	28.19a	45.14a
	6	9.76c	51.11a	40.85a	43.47a	25.62a	49.81a
	7	13.33c	29.67bc	43.33a	48.33a	28.00a	49.67a
	LSD	11.84	8.75	16.08	41.08	22.55	6.42

Means with different letters are significantly (*p* < 0.05) different across the column in each section. LSD: least significant difference.

## Discussion

Most phylogenetically related strains were pathogens that have been previously isolated from various plants. Strains F2B and 296 were most closely associated with *Fusarium* sp. MH605341, is a pathogen associated with the root of a popular medicinal plant, *Panax ginseng* from Changchun, China. This strain was recently matched with high similarity to an endophyte isolated from Orchid by Yokoya et al. (2021). These two strains (*Fusarium tricinctum* Strain 294 and *Fusarium proliferatum* Strain 296) also showed high similarity with *Fusarium* sp. Strains MN256656 and MT561397. Strain MT561397 was a recently isolated strain by Langer and Bußkamp (2021) as a pathogen from the bark of the European beech (*Fagus sylvatica*) a deciduous tree belonging to the beech family Fagaceae commonly refers to as beech. The two *T. virens* TC4 and TC7 *Trichoderma* strains that expressed the most similar phylogenetic relationship in this study had related clades with TMS1, TMZ2, TC8 and, TC6. These strains all together showed a phylogenetic relationship with other strains that were recently confirmed as potential bioagents. They included *T. gamsii* MT090014 a *Trichoderma* strainTY190-12 recently isolated from QinLing Mountain, in China by An et al. (2020), and *T. koningiopsis* KC481706 a newly isolated *Trichoderma* endophyte of deciduous wood tree *Leucas aspera* strain. *T. koningiopsis* KC481706 is a *Trichoderma* strain UFSM T002sB recently isolated by Tseng et al. (2020). Interestingly, UFSM T002sB was demonstrated to promote the growth of *Arabidopsis thaliana* and *Nicotiana attenuata* against pathogen attacks by Tseng et al. (2020). In another view, strain TMZ1 was distinct from others which was more phylogenetically different from other strains, it showed high similarity with *T. koningiopsis* KC481706 and *T. gamsii* MT090014. *T. gamsii* MT090014 is another *Trichoderma* strain TY190-12 also isolated by An et al. (2020) from China. Strain TMZ1 also shared a high similarity with *Hypocrea koningiopsis* EU076928. EU076928 is a soil bioagent strain 16-M-1 recently isolated from agricultural soil in Vietnam by Ghazanfar et al. (2018).

Investigation into the biocontrol potential of *Trichoderma* species against pathogens of crops of agricultural importance was inspired by the need to develop viable biologically based pesticides, which are eco-friendly to replace synthetic pesticides (Olowe et al., 2020; Akanmu et al., 2021). Variations in the inhibitory potentials of different *Trichoderma* species was demonstrated in this study as each *Trichoderma* species expressed the highest inhibition of specific *Fusarium* species such as the efficacy of TMZII against OakF2B (31.17%), TCVIII against Fus 294 (45.18%) and TCIV against Oak F3 (44.15%). This result could be ascribed to their varying sources and attributes of the organisms, an earlier investigation conducted by Anjum et al. (2020) also reported a similar observation, where five different *Trichoderma* species evaluated showed disparities in the inhibition of *Fusarium oxysporum* f. spp. *capsica* causing *Fusarium* wilt of Chili. More so, the respective order of antagonistic activities of *Trichoderma* species with TMZII as the most effective is consistent with some earlier reports on the inhibitory potentials of *Trichoderma* species against plant pathogens (Talapatra et al., 2017; Olowe et al., 2022). Furthermore, advantages recorded in the use of *Trichoderma* are justified by their rapid establishment within the stable microbial communities, and control of pathogenic, competitive, or deleterious microflora through various mechanisms (Vinale et al., 2008).

The results similar to that of the dual culture assay were obtained from the *Trichoderma* culture filtrates, especially in TCIV, TMSI, and TCVII across the *Fusarium* species. Although, variations were recorded in the antagonistic activities of the culture filtrates of each specie with TCIV expressing the highest percentage inhibition against Oak F3 (52.39%), and Oak F2B (48.54%), Fus 294 (46.65%), and Fus 296 (44.48%). Similar results with respect to the genetic variations among the *Trichoderma* species had earlier been reported (Rai et al., 2016). More so, the variation in the virulence of *Fusarium* species could contribute to the differences in the rate of inhibition recorded by the *Trichoderma* species (Kolp et al., 2018). This affirms the varying levels of biocontrol activities against pathogens, as also reported by Nandini et al. (2021). However, these results validate the earlier findings of the dual culture and affirm the antagonistic potentials of *Trichoderma* species through different mechanisms of action, including competition for nutrients, mycoparasitism, and antibiosis (Rajani et al., 2021). This strongly underscores the mycoparasitic potential of these *Trichoderma* species against pathogens such as *Fusarium* species (Olowe et al., 2020), as also affirmed in the interactions of TMZII against *Fus* 294 and 296 in this study. While the type of interaction observed between the *Fusarium* and *Trichoderma* was not verified, this study suggests the existence of antifungal traits in the culture filtrates, and the suspected mycoparasitism is found in agreement with the report of Mukherjee et al. (2022) as the most effective disease suppression method.

## Conclusion

The current investigation verified the effectiveness of certain *Trichoderma* isolates as bioagents against *Fusarium* pathogens of oak and rice. The *Trichoderma* species in both dual and culture filtrate evaluation demonstrated varying degrees of inhibitory effects against the *Fusarium* pathogens of rice and oak leaves, thereby ascertaining the antagonistic potentials of indigenous *Trichoderma* species against *Fusarium* pathogens. This research identifies more members of the genus *Trichoderma* with promising antagonistic prowess against fungi of economic importance, such as *Fusarium* species. Further experiments in the field are however imperative before credible assertions could be made on their mycoparasitic capabilities.

## Data availability statement

The datasets presented in this study can be found in online repositories. The names of the repository/repositories and accession number(s) can be found in the article/supplementary material.

## Author contributions

All authors listed have made a substantial, direct, and intellectual contribution to the work and approved it for publication.

## Funding

OB recognizes National Research Fund (NRF South Africa) for grants (UID123634 and UID132595) that support work in her research group.

## Conflict of interest

The authors declare that the research was conducted in the absence of any commercial or financial relationships that could be construed as a potential conflict of interest.

## Publisher’s note

All claims expressed in this article are solely those of the authors and do not necessarily represent those of their affiliated organizations, or those of the publisher, the editors and the reviewers. Any product that may be evaluated in this article, or claim that may be made by its manufacturer, is not guaranteed or endorsed by the publisher.

## Footnotes

^1^ https://blast.ncbi.nlm.nih.gov/
